# Work for the Wounded of the Allied Armies

**Published:** 1914-12-19

**Authors:** 


### Work for the Wounded of the Allied Armies.

(From our Special Correspondents.)

### CARDIFF: SOLDIERS' PRAISE FOR THE TRANSPORT ARRANGEMENTS.

Another batch of wounded and sick soldiers, numbering 120, arrived in Cardiff on December 11, in charge of Captain Bird. This was the second contingent in five days. The majority of the men had been brought direct from the trenches in France, and the condition of their clothing and the fact that many of them suffered from frostbite indicated the severity of the weather. They were full of praise for the admirable manner in which they had been conveyed direct from the fighting line in the face of danger and difficulty. Major Ewen Maclean, assisted by Captain W. Nicholson, superintended the detraining arrangements and the distribution to the hospitals. Commandant Owen Davies was in charge of the Ambulance Detachments, and Major Jordan had control of the motor transport.

Amongst those who witnessed the arrival of the soldiers was Mr. Donald Maclean, M.P., Deputy Chairman of the House of Commons, and brother of Major Maclean. There were 42 "cot" cases, and the allocation was: to Howard Gardens, 30; Splott Road, 30; Albany Road, 60.

### CHRISTMAS IN THE BASE HOSPITALS.

Information has been received at the 3rd Western General Hospital, Cardiff, from the Directors of the Princess Mary Christmas Fund, to the effect that every wounded soldier in hospital on Christmas Day will receive a personal present from the Fund. All the men who from December 12 until Christmas leave the hospital on furlough will have their gifts sent to their homes.

### ST. BARTHOLOMEW'S HOSPITAL, ROCHESTER.

Two batches of wounded British soldiers, numbering about 350, have been received in the Chatham District during the past fortnight. The first batch of about 200 were men very badly wounded, and in some cases suffering from frostbite. The second batch were principally sick cases. All the patients were dealt with promptly on arrival at Chatham Station by the Transport Service Corps of private motor vehicles and motor ambulances, and conveyed to the various hospitals in the Chatham area.

### DONCASTER: FIRST WOUNDED SOLDIERS ARRIVE.

Doncaster received its first contingent of wounded warriors last week—not serious cases direct from the Front, but convalescent cases transferred from the base hospital at Sheffield. Through the generosity of Mr. W. S. Arnold, contractor, who recently left the town, his late residence in Thorne Road has been placed at the disposal of the V.A.D., and accepted by the War Office as a convalescent hospital; and it was here that the twenty soldiers from Sheffield were brought. Edenfield, as the house is called, has received the name of the "Arnold" Auxiliary Hospital as a compliment to its generous owner. The house stands in large grounds and shrubberies, with a paddock in which the convalescents can take active exercise. The rooms are spacious, and a large room on the ground floor, now furnished as a ward with seven beds, is heated by the same pipes that supply the neighbouring conservatories. Altogether there are twenty-five beds available. The hospital is under the superintendence of Sister Pease, who is the matron of a nursing home close by. The Commandant of the detachment is Mrs. W. H. Pickering, widow of the late Divisional Inspector of Mines, hero of the Cadeby Colliery disaster. Miss N. A. Smith combines the offices of hon. secretary and quartermaster. The hospital can draw upon the services of over fifty qualified St. John Ambulance nurses, and these are worked in relays, two being on duty in the hospital at night, in readiness for any sudden call. The whole of the cooking and "housework" is done by the V.A.D. members themselves. The medical superintendent is Surgeon-Colonel Stevenson, who receives ready assistance, as required, from other local practitioners. Loversall, a little village a mile or two out of Doncaster, boasts a Voluntary Aid Detachment occupied in staffing an Ambulance Rest Station at the Doncaster Railway Station. Now some sixteen convalescent soldiers have been sent down to the auxiliary hospital in Loversall Hall, the residence of the Commandant, Mrs. F. G. Skipwith. The hospital is under the superintendence of Miss Ross, of Wadworth Hall.

### SHEFFIELD: OVER 2,000 SOLDIERS TREATED.

Early on December 10 another 120 more or less convalescent soldiers arrived at Sheffield. Two things struck the onlooker. First, the improvement in dealing with the arrivals since the first batch came to Sheffield; the work is completed in half the time, for the number of ambulances has been trebled. The second point was the large majority of cases of sickness which are coming from the Front. The eleventh convoy includes a number of "sick" cases, and not a few of them men who have suffered severely from the frosty weather. This is not to be wondered at. One man informed our Sheffield correspondent that he was in the trenches ten days, standing in mud and water, and when he came out he had to tramp several miles through snow and slush. These conditions account for the large number of men who are having to come home for rest and change. They do not stay long in hospital. They are sent to convalescent homes, quite a number of which the officer commanding the 3rd Northern General Hospital has secured among the beautiful scenes of Derbyshire or the moorland of Yorkshire. Including Territorials, the hospital staff have dealt with over 2,000 cases of wounds and sickness since the commencement of the war.

